# Bone Adaptations to a Whole Body Vibration Protocol in Murine Models of Different Ages: A Preliminary Study on Structural Changes and Biomarker Evaluation

**DOI:** 10.3390/jfmk10010026

**Published:** 2025-01-10

**Authors:** Ida Cariati, Roberto Bonanni, Cristian Romagnoli, Lucio Caprioli, Giovanna D’Arcangelo, Virginia Tancredi, Giuseppe Annino

**Affiliations:** 1 Department of Systems Medicine, “Tor Vergata” University of Rome, 00133 Rome, Italy; ida.cariati@uniroma2.it (I.C.); giovanna.darcangelo@uniroma2.it (G.D.); tancredi@uniroma2.it (V.T.); g_annino@hotmail.com (G.A.); 2 Department of Biomedicine and Prevention, “Tor Vergata” University of Rome, 00133 Rome, Italy; 3 Department of Human Science and Promotion of Quality of Life, San Raffaele Open University, 00166 Rome, Italy; cristian.romagnoli@uniroma5.it; 4 Sports Engineering Laboratory, Department of Industrial Engineering, “Tor Vergata” University of Rome, 00133 Rome, Italy; lucio.caprioli@uniroma2.it; 5 Centre of Space Bio-Medicine, “Tor Vergata” University of Rome, 00133 Rome, Italy

**Keywords:** whole body vibration, bone, musculoskeletal system, physiology, aging, sedentariness, exercise, training, biomarkers, vibratory training

## Abstract

**Background/Objectives**: Whole body vibration (WBV) is a valuable tool to mitigate physiological adaptations related to age and inactivity. Although significant benefits have been found at the musculoskeletal level, including increased bone mass and reduced muscle atrophy, the underlying biological mechanisms remain largely unknown. Therefore, our study aimed to evaluate the effects of vibratory training on bone tissue in murine models of different age groups by investigating the structural and distribution changes in some crucial biomarkers involved in musculoskeletal homeostasis. **Methods**: Specifically, 4-, 12-, and 24-month-old mice were trained with a WBV protocol characterized by three series of 2 min and 30 s, interspersed with a recovery period of the same duration, on a 3-weekly frequency for 3 months. At the end of the training, histological and morphometric analyses were conducted, in association with immunohistochemical analysis to investigate changes in the distribution of fibronectin type III domain-containing protein 5 (FNDC5), NADPH oxidase 4 (NOX4), and sirtuin 1 (SIRT1). **Results**: Our preliminary results showed that WBV improves musculoskeletal health by preserving bone architecture and promoting up-regulation of FNDC5 and SIRT1 and down-regulation of NOX4. **Conclusions**: Our study confirms vibratory training as a viable alternative to counter musculoskeletal decline in elderly and/or sedentary subjects. Further investigations should be conducted to deepen knowledge in this field and explore the role of other molecular mediators in physiological adaptations to vibration.

## 1. Introduction

The musculoskeletal system is continuously undergoing remodeling processes that ensure its functionality over time, providing mobility, stability, and good quality of life [1]. However, intrinsic and extrinsic factors, including hormonal variations, nutritional deficiencies, and physical activity levels, profoundly affect its structural and molecular integrity [2]. Also, the aging process is associated with numerous alterations in the musculoskeletal system both in structural terms, such as reduced bone mineral density (BMD) and increased muscle fiber atrophy, and in molecular terms, including altered expression patterns of biomarkers that regulate bone and muscle growth [3].

Fortunately, regular exercise promotes and safeguards musculoskeletal health, counteracting age-related degenerative diseases, such as osteoporosis and sarcopenia, and mitigating aging damage [4]. Interestingly, the controlled application of mechanical vibration has been shown to bring about physiological improvements analogous to physical exercise, suggesting the possibility of developing specific vibratory training protocols for subjects who are sedentary or generally unable to exercise [5]. In this context, numerous pieces of evidence have reported the benefits of whole body vibration (WBV) in both human and murine models, highlighting the effectiveness of this form of passive exercise in promoting physical and mental health [6,7,8]. Particularly, Cariati et al. have recently shown significant structural improvements in both nervous and musculoskeletal systems in 4-month-old mice subjected to 3-weekly vibratory training for 3 months, observing more significant benefits with the application of a protocol characterized by a low vibration frequency, a shorter vibration exposure period, and a more extended recovery period between two sessions [9]. These results agree with those previously reported by the same authors, as demonstrated by the increase in the diameter of muscle fibers, the complete preservation of the sarcomeric structure, and the number of mitochondria in a middle-aged mouse model subjected to vibratory training [10].

Although the beneficial effects of WBV are now well documented, evidence on biomarkers involved in physiological adaptations to vibratory training is limited and mainly focuses on mediators such as fibronectin type III domain-containing protein 5 (FNDC5), irisin, and peroxisome proliferator-activated receptor γ coactivator transcription factor 1α (PGC1-α) [11,12]. However, other mediators, such as sirtuin 1 (SIRT1) and NADPH oxidase 4 (NOX4), could play a significant role, considering their involvement in the effects of exercise on health status [13,14]. Particularly, FNDC5 may be among the main contributors to the musculoskeletal effects of WBV [9]. The expression of this protein and the polypeptide released from its cleavage, irisin, increases significantly in muscle, bone, and nerve tissue, as well as in adipose tissue, in response to exercise [15,16,17]. In this regard, Kim et al. (2018) investigated potential targets for mechanistic regulation of irisin, identifying integrin αV/β5 as a downstream receptor for irisin [16]. This myokine is known to stimulate osteoblast proliferation and differentiation mainly through the mitogen-activated protein kinase (MAPK), AMP-activated protein kinase (AMPK), and phosphoinositide 3-kinase (PI3K)/AKT pathways, representing a promising therapeutic strategy against osteoporosis [18]. Furthermore, the serum increase in irisin was found by Greulich and colleagues (2014), who conducted a randomized clinical trial in patients with chronic obstructive pulmonary disease undergoing WBV in combination with conventional physiotherapy. Interestingly, patients undergoing WBV showed a significant increase in the expression of PGC1-α, which stimulates FNDC5 expression and, consequently, the circulating levels of irisin [11].

SIRT1, a nicotinamide adenine dinucleotide (NAD)-dependent deacetylase, is also known to play a crucial role in exercise effects, ranking as a potential biomarker responsible for physiological adaptations to WBV [19]. In this regard, Li et al. found increased mRNA expression of SIRT1 and PGC1-α in 18-month-old male Sprague–Dawley rats trained 5 days a week for 8 weeks on a treadmill, as well as a reduction in apoptosis, suggesting a role in counteracting age-related sarcopenia [20]. The benefits of SIRT1 have also been described in bone tissue, given its ability to reduce the expression of sclerostin, the primary negative regulator of bone growth [21], suppress osteoclastogenesis [22], and reduce age-related cartilage degeneration, representing a potential therapeutic target to counter osteoporosis and osteoarthritis [13,23]. Noteworthy, SIRT1 is also involved in the regulation of energy homeostasis, as it reduces obesity, promotes fat mobilization, and enhances mitochondrial metabolism, as well as increases the differentiation capacity of bone marrow stromal cells (BMSCs), promoting osteoblastogenesis [24,25]. In this regard, Cohen-Kfir and colleagues (2011) observed a significant reduction in bone mass and upregulated adipogenesis in the bone marrow of *SIRT1* haplo-insufficient (SIRT1^+/−^) female mice, suggesting a crosstalk between metabolic organs and bone and proposing SIRT1 as a potential therapeutic target for the prevention of metabolic diseases and the development of osteoporosis [26,27].

Interestingly, the beneficial action of SIRT1 on the musculoskeletal system could be expressed through oxidative stress suppression and activation of antioxidant pathways [28]. In this regard, Dasgupta and colleagues highlighted the ability of SIRT1 to improve muscle atrophy by regulating NOX4 in tumor-bearing mice [29]. NOX4 is an essential inducer of oxidative stress, being able to produce superoxide ion (O_2_^−^) and hydrogen peroxide (H_2_O_2_) in various pathological conditions, including age-related sarcopenia [30,31,32]. Furthermore, high levels of NOX4-dependent reactive oxygen species (ROS) have also been found to be responsible for age-related bone loss, suggesting NOX4 as a potential therapeutic target to counteract osteoporosis during aging [33]. Although the influence of vibratory training on NOX4 activity has not yet been investigated, some alterations in its expression have been reported in response to exercise [34,35]. Specifically, Qi et al. (2020) demonstrated that an 8-week swimming exercise reduced NOX4 expression and increased AMPK expression in skeletal muscle of a mouse model of insulin resistance (IR)-induced high-fat diet, preventing ROS production and enhancing PI3K/AKT signal transduction [36].

Based on this evidence, modulating crucial biomolecules such as FNDC5, NOX4, and SIRT1 by WBV could be a promising therapeutic approach to counteract the progression of age-related musculoskeletal disorders in subjects restricted to a sedentary lifestyle. Therefore, the goal of this study, which represents the continuation of our previous research [9,10,37], was to investigate the effects of a WBV protocol, appropriately designed in terms of frequency, duration, and recovery interval between sessions, in murine models of three different age groups by evaluating structural changes in bone tissue of 4-month-old young mice, 12-month-old adult mice, and 24-month-old aged mice. Specifically, this research aimed to (i) evaluate structural changes in bone tissue induced by the WBV protocol by histological and morphometric analysis, characterizing any changes that might reflect adaptation or degeneration processes related to both age and vibration exposure, and (ii) study any alterations in the distribution patterns of FNDC5, NOX4, and SIRT1, all biomarkers potentially involved in physiological adaptations to WBV.

## 2. Materials and Methods

### 2.1. Animals

Male mice of the wild-type BALB/c strain were used in this study following approval of the experimental protocols by the Ministry of Public Health (authorization no. 86/2018-PR) and following the procedures established by the European Union Council Directive 2010/63/EU for animal experiments [38]. In total, 45 mice divided into age groups were used (Figure S1): 4-month-old young mice (n = 15), 12-month-old adult mice (n = 15), and 24-month-old aged mice (n = 15). For each age group, we considered an intervention group with mice subjected to a specific WBV protocol (n = 5) and a control group (CTRL) consisting of mice subjected to the same regimen of placement on the box in the platform, the same environmental exposure including motor sounds, but not exposed to vibratory training (n = 5). An additional control group (SED) for each age group consisting of sedentary mice not subjected to any training (n = 5) was included. Although both control groups underwent the same assessments, the data reported are representative of the CTRL group to avoid redundancies due to the almost complete overlap in results (Figures S2–S4).

The same housing and feeding conditions were adopted for each animal, subjecting them to daily checks by resident veterinarians and experimenters to monitor their physical condition, including hair and skin, weight, and body functions. Furthermore, all animals included in this study did not engage in any kind of physical activity prior to the start of the experimental protocol.

### 2.2. Vibratory Training Protocol

A vibrating platform (Power Club, Vigarano Mainarda, 44049 FE, Italy) was used to subject animals to mechanical vibrations. Its characteristics included a power supply of 220 V, a total maximum electrical power of 0.12 kW, and a vertical-type vibration, with a vibration frequency of 45 Hz, an acceleration of 2 g, and an amplitude of 1.5 mm.

The training protocol consisted of 3 vibration series of 2 min and 30 s each, interspersed with 2 min and 30 s of recovery. In total, 36 training sessions were conducted, with a distribution over 12 weeks, 3 times a week. The animals were raised on a light–dark cycle of 12:12 h, and training was carried out in the morning, between 10 and 11 a.m, to minimize the influence of circadian rhythms on performance and avoid fluctuations associated with sleep and wake periods, which could affect their response to exercise. All sessions were conducted in controlled environmental conditions, minimizing ambient noise. Finally, the equipment used for the experimental protocol was regularly checked to ensure that there were no technical variations that could influence the results.

### 2.3. Histological and Morphometric Analysis

All animals, sedentary and trained, were sacrificed at the end of the experimental period under anesthesia with halothane (2-bromo-2-chloro-1,1,1-trifluor-ethane) to minimize their suffering, according to experimental procedures [39]. Immediately after sacrifice, samples of bone tissue from the lumbar segment of the spine were taken from each animal to conduct histological and morphometric analyses. All samples were fixed in 4% paraformaldehyde for 24 h and then washed with distilled water to start the decalcification process. Particularly, the samples were immersed in the decalcifying solution (05-M03004, Bio-Optica, Milan, Italy) at room temperature under continuous stirring. The decalcification process was completed when the bone was penetrable by a needle without any force. Subsequently, the samples were washed abundantly with phosphate-buffered saline (PBS) to remove acid residues, dehydrated and finally embedded in paraffin. To perform morphological analysis, 3 μm-thick sections were stained with hematoxylin and eosin (H&E) (Bio-Optica, Milan, Italy). H&E slides were visualized by a Nikon upright microscope ECLIPSE Ci-S (Nikon Corporation, Tokyo, Japan) connected to a Nikon digital camera. Images were acquired at 20× magnification using NIS-Elements software (5.30.01; Laboratory Imaging, Prague, Czech Republic).

Two blinded observers performed morphometric analysis of bone tissue, evaluating bone morphometric parameters, such as bone volume (BV/TV), trabecular thickness (Tb.Th), and trabecular separation (Tb.S). For each parameter, a reference area was set using NIS-Elements software, so the size of the region of interest was the same regardless of the type of evaluation performed. Specifically, BV/TV was calculated as the ratio of trabecular bone volume (BV) to the total volume of the analyzed region (TV), expressed as a percentage. Tb.Th was determined by measuring the mean thickness of the bone trabeculae, calculated as the mean distance between the opposite edges of each trabeculae, while Tb.S was calculated as the mean distance between adjacent trabeculae within the analyzed region. In all cases, 8 non-overlapping readings were conducted for each experimental animal, and measurements were performed at 40× magnification.

### 2.4. Immunohistochemistry

Immunohistochemical analysis investigated FNDC5, NOX4, and SIRT1 immunolocalization in the experimental samples. Briefly, 3 μm-thick sections were pretreated with ethylenediaminetetraacetic acid (EDTA) citrate (pH 6.0) for 20 min at 95 °C and then incubated with rabbit polyclonal anti-FNDC5 C-terminal (dilution 1:100; ab181884, AbCam, Cambridge, UK), rabbit polyclonal anti-NOX4 (dilution 1:100; NB110-58849, Novus Biologicals, Centennial, CO, USA), and mouse monoclonal anti-SIRT1 [19A7AB4] (dilution 1:100; ab110304, AbCam, Cambridge, UK) for 1 h at room temperature. Washings were performed with PBS/Tween20 (pH 7.6) (UCS Diagnostic, Rome, Italy), and reactions were revealed by the Horseradish Peroxidase-3,3-diaminobenzidine (HRP-DAB) Detection Kit (UCS Diagnostic, Rome, Italy). Specifically, 50 μL DAB/450 μL of substrate was incubated for 3 min. To assess the background of immunostaining, we included negative controls for each reaction (Figure S4) by incubating the sections with secondary antibodies (HRP) and a detection system (DAB).

The distribution levels of FNDC5, NOX4, and SIRT1 were quantified using mean optical density (MOD), a semi-quantitative method that provides a measure of signal intensity in relation to the amount of protein present in tissue sections. Specifically, NIS-Elements software (5.30.01; Laboratory Imaging, Prague, Czech Republic) was used to analyze the images, identifying areas of interest where the chromogenic signal was present. The MOD was calculated by measuring the signal intensity in these areas, reflecting the relative concentration of the protein for each sample analyzed. For each condition, the experiment was conducted in triplicate (n = 15 from N = 5 experiments).

### 2.5. Statistical Analysis

Statistical analysis was performed using GraphPad Prism 8 software (GraphPad Prism 8.0.1, La Jolla, CA, USA). For weight assessments, two-way ANOVA was used, as the comparison between the control group and the trained group was carried out in three time periods of the experimental protocol. Then, Sidak’s multiple comparison test was applied to determine the significant differences between the groups at each time while maintaining a reliable level of significance. In addition, the calculation of Eta squared (η^2^) was performed to quantify the magnitude of main effects and interactions [40]. Morphometric and immunohistochemical data with normal distribution were compared by unpaired *t*-test with Welch’s correction, as the comparison was made between two independent groups: the control group and the trained group. All data were expressed as mean ± standard error and were considered significantly different if *p* < 0.05.

## 3. Results

### 3.1. WBV Increases Body Weight in Murine Models of Different Age Groups

**Figure 1:**
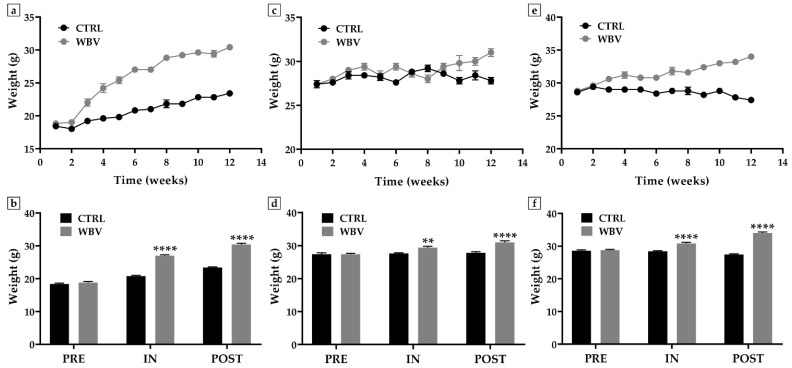
Effects of a whole body vibration (WBV) protocol on weight in murine models of different age groups. (**a**,**b**) The most significant weight changes were measured in 4-month-old young mice, with a significant increase in mean weight at the 6 weeks of training (IN phase) and at the end of training (POST) (**** *p* < 0.0001). (**c**,**d**) WBV promoted a significant increase in body weight in 12-month-old adult mice in the IN (** *p* < 0.01) and POST (**** *p* < 0.0001) phases compared with sedentary animals. (**e**,**f**) Significant weight changes were induced by vibratory training in 24-month-old mice in the IN (**** *p* < 0.0001) and POST (**** *p* < 0.0001) phases.

The weight of the mice was monitored throughout the experimental period to investigate any effects induced by vibratory training. Specifically, Figure 1 shows the weight changes measured from week 1 to week 12 in association with the comparison of the mean body weight values at the beginning (PRE), middle (IN), and end (POST) of the study protocol.

Importantly, WBV promoted a significant increase in body weight in all experimental groups, with more or less marked variations according to age group. Not surprisingly, more pronounced weight changes were observed in 4-month-old young mice. The mean weight was 18.4 ± 0.2 g in the CTRL group and 18.8 ± 0.4 g in the WBV group in the PRE phase (*p* = 0.74), whereas the IN phase was characterized by mean weight values of 20.8 ± 0.2 g in the CTRL group and 27.0 ± 0.3 g in the WBV group (*p* < 0.0001). A significant increase in weight was detected after 12 weeks of training, with values of 23.4 ± 0.2 g in the CTRL group and 30.4 ± 0.4 g in the WBV group (*p* < 0.0001). The two-way ANOVA showed significant main effects of the time factor [F(2, 24) = 378.5, η^2^ = 0.606, *p* < 0.0001], experimental group [F(1, 24) = 330.3, η^2^ = 0.264, *p* < 0.0001], and a significant interaction between time and group [F(2, 24) = 69.5, η^2^ = 0.111, *p* < 0.0001] (Figure 1a,b).

In the 12-week-old adult mice, no weight change was found in the sedentary animals; however, significant weight gain was found as early as week 6 of training. Specifically, the mean weight was 27.4 ± 0.4 g in the CTRL group and 27.4 ± 0.2 g in the WBV group in the PRE phase (*p* > 0.99), while mean weight values of 27.6 ± 0.2 g in the CTRL group and 29.0 ± 0.4 g in the WBV group were measured in the IN phase (*p* < 0.01). A significant increase in weight was observed at the end of the study protocol, with values of 27.8 ± 0.4 g in the CTRL group and 31.0 ± 0.4 g in the WBV group (*p* < 0.0001). The results of the two-way ANOVA indicate that both the time factor [F(2, 24) = 15.44, η^2^ = 0.289, *p* < 0.0001] and the experimental group [F(1, 24) = 32.05, η^2^ = 0.300, *p* < 0.0001] have significant main effects on weight. In addition, a significant interaction was observed between these two factors [F(2, 24) = 9.897, η^2^ = 0.186, *p* = 0.0007] (Figure 1c,d).

Finally, vibratory training also promoted a significant weight increase in the 24-month-old mice, with values of 27.4 ± 0.2 g in the CTRL group and 34.0 ± 0.3 g in the WBV group (*p* < 0.0001) at week 12. On the other hand, the mean weight was 28.6 ± 0.2 g in the CTRL group and 28.8 ± 0.2 g in the WBV group in the PRE phase (*p* = 0.94), whereas mean weight values of 28.4 ± 0.2 g in the CTRL group and 30.8 ± 0.4 g in the WBV group were found in the IN phase (*p* < 0.0001). Statistical analysis showed that both the time factor [F(2, 24) = 26.17, η^2^ = 0.131, *p* < 0.0001] and the experimental group [F(1, 24) = 184.0, η^2^ = 0.462, *p* < 0.0001] contributed significantly to the observed variability. A significant interaction between the two factors also emerged [F(2, 24) = 68.96, η^2^ = 0.346, *p* < 0.0001] (Figure 1e,f).

### 3.2. WBV Exposure Improves Bone Tissue Architecture

Histological analysis revealed distinctive features of trabecular bone in all experimental groups, confirmed by the measurement of conventional bone morphometric parameters. Among them, bone volume (BV/TV), trabecular thickness (Tb.Th), and trabecular separation (Tb.S) were used as key indicators to assess bone tissue quality.

**Figure 2:**
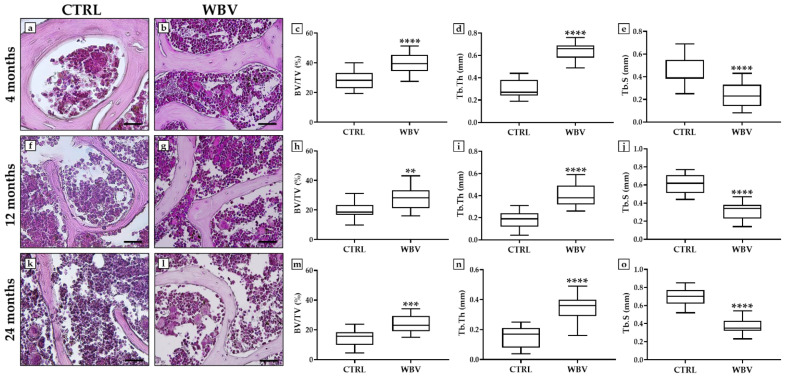
Hematoxylin and eosin (H&E) sections of lumbar segment of the spine from all experimental groups and measurement of significant bone morphometric parameters. (**a**–**e**) The 4-month-old young mice in the WBV group showed the highest values of bone volume (BV/TV) and trabecular thickness (Tb.Th), in association with reduced space between bone trabeculae (Tb.S), compared with sedentary animals (**** *p* < 0.0001). (**f**–**j**) In 12-month-old adult mice, WBV significantly increased BV/TV (** *p* < 0.01) and Tb.Th (**** *p* < 0.0001), as well as significantly reduced Tb.S (*p* < 0.0001). (**k**–**o**) WBV also improved bone architecture in 24-month-old mice, promoting a significant increase in BV/TV (*** *p* < 0.001) and Tb.Th (**** *p* < 0.0001) and a significant reduction in Tb.S (**** *p* < 0.0001). For 20× images, the scale bar represents 100 μm. For each condition, the experiment was conducted in triplicate (n = 15 from N = 5 experiments).

Interestingly, vibratory training improved the structural organization of bone tissue, with significant changes in the measured parameters depending on the age of the experimental mice. In the 4-month-old young mice, there was a marked increase in BV/TV and Tb.Th was observed, accompanied by a reduction in Tb.S. The BV/TV values were 28.2 ± 1.5 for the CTRL group and 39.4 ± 1.9 for the WBV group (*p* < 0.0001), while the Tb.Th values were 0.29 ± 0.02 for the CTRL group and 0.63 ± 0.02 for the WBV group (*p* < 0.0001). In contrast, the Tb.S values were 0.45 ± 0.03 for the CTRL group and 0.23 ± 0.03 for the WBV group (*p* < 0.0001) (Figure 2a–e).

In the 12-month-old adult mice, structural improvement was detected, but with a less pronounced increase in BV/TV and Tb.Th than in younger mice. Specifically, the BV/TV values were 19.7 ± 1.5 for the CTRL group and 28.1 ± 1.9 for the WBV group (*p* < 0.01), while the Tb.Th values were 0.18 ± 0.02 for the CTRL group and 0.39 ± 0.02 for the WBV group (*p* < 0.0001). On the other hand, the Tb.S values were 0.61 ± 0.03 for the CTRL group and 0.32 ± 0.02 for the WBV group (*p* < 0.0001) (Figure 2f–j).

Even in the 24-month-old mice, WBV exposure significantly increased BV/TV and Tb.Th and significantly decreased Tb.S, although aging may attenuate the impact of vibratory training on bone tissue. The BV/TV values were 14.6 ± 1.4 for the CTRL group and 24.1 ± 1.6 for the WBV group (*p* < 0.001), while the Tb.Th values were 0.15 ± 0.02 for the CTRL group and 0.35 ± 0.02 for the WBV group (*p* < 0.0001). Finally, the Tb.S values were 0.69 ± 0.02 for the CTRL group and 0.37 ± 0.02 for the WBV group (*p* < 0.0001) (Figure 2k–o).

### 3.3. FNDC5, NOX4, and SIRT1 Immunolocalization Is Modulated by WBV Exposure

An immunohistochemistry analysis was performed to detect changes in the distribution of FNDC5, NOX4, and SIRT1 in bone tissue after WBV exposure.

**Figure 3:**
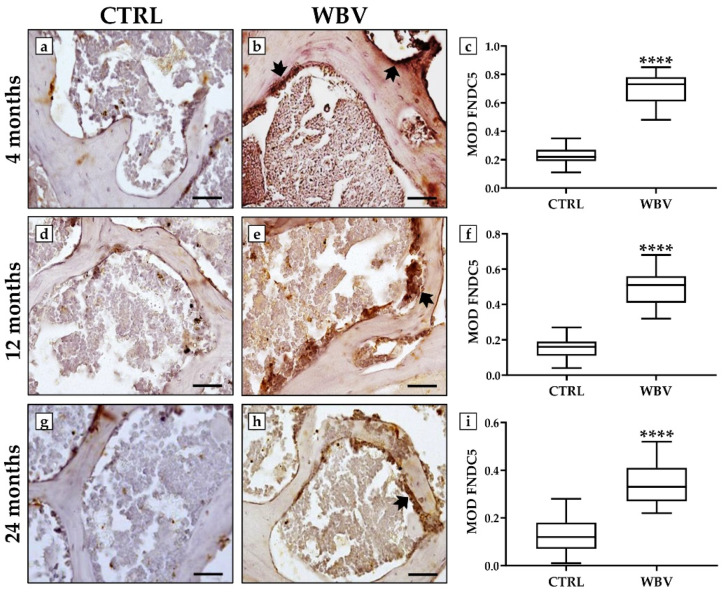
Evaluation of fibronectin type III domain-containing protein 5 (FNDC5) immunolocalization in bone tissue of young, adult, and aged mice by immunohistochemistry analysis. (**a**–**c**) The highest levels of FNDC5 (arrows) were measured in young, trained mice, with a significant increase in FNDC5 distribution compared with the CTRL group (**** *p* < 0.0001). (**d**–**f**) A significant increase in FNDC5 distribution (arrow) was observed in 12-month-old adult mice exposed to WBV compared with sedentary animals (**** *p* < 0.0001). (**g**–**i**) FNDC5 levels (arrow) were significantly increased in the bone tissue of 24-month-old mice after WBV exposure compared with the CTRL group (**** *p* < 0.0001). For 20× images, the scale bar represents 100 μm. For each condition, the experiment was conducted in triplicate (n = 15 from N = 5 experiments).

Figure 3 shows a significant increase in FNDC5 in the bone tissue of all trained animals, with an age-related distribution pattern. Indeed, in the 4-month-old young mice, the MOD values for FNDC5 were 0.22 ± 0.02 in the CTRL group and 0.69 ± 0.03 in the WBV group (*p* < 0.0001). Similarly, in the 12-month-old adult mice, the MOD values for FNDC5 were 0.15 ± 0.02 in the CTRL group and 0.49 ± 0.03 in the WBV group (*p* < 0.0001). Finally, in the 24-month-old mice, the MOD values for FNDC5 were 0.12 ± 0.02 in the CTRL group and 0.34 ± 0.02 in the WBV group (*p* < 0.0001).

**Figure 4:**
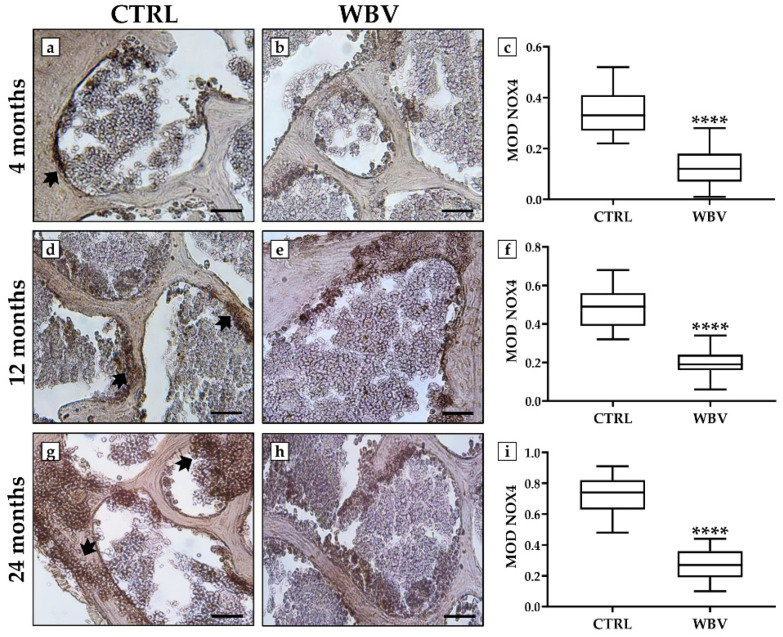
Evaluation of NADPH oxidase 4 (NOX4) immunolocalization in bone tissue of young, adult, and aged mice by immunohistochemistry analysis. (**a**–**c**) A significant reduction in NOX4 distribution (arrow) was observed in 4-month-old young mice exposed to WBV compared with sedentary animals (**** *p* < 0.0001). (**d**–**f**) NOX4 levels (arrow) were significantly reduced in the bone tissue of 12-month-old adult mice after WBV exposure compared with the CTRL group (**** *p* < 0.0001). (**g**–**i**) The highest NOX4 levels (arrows) were measured in 24-month-old sedentary mice, while protein distribution was significantly reduced in the WBV group of the same age group (**** *p* < 0.0001). For 20× images, the scale bar represents 100 μm. For each condition, the experiment was conducted in triplicate (n = 15 from N = 5 experiments).

Interestingly, WBV exposure significantly reduced the distribution of NOX4, a known inducer of oxidative stress, while the highest levels of this marker were found in the 24-month-old CTRL group (Figure 4). In fact, in the 4-month-old young mice, the MOD values for NOX4 were 0.33 ± 0.02 in the CTRL group and 0.13 ± 0.02 in the WBV group (*p* < 0.0001), whereas in the 12-month-old adult mice, the MOD values for NOX4 were 0.47 ± 0.03 in the CTRL group and 0.19 ± 0.02 in the WBV group (*p* < 0.0001). Finally, in the 24-month-old mice, the MOD values for NOX4 were 0.73 ± 0.03 in the CTRL group and 0.28 ± 0.02 in the WBV group (*p* < 0.0001).

**Figure 5:**
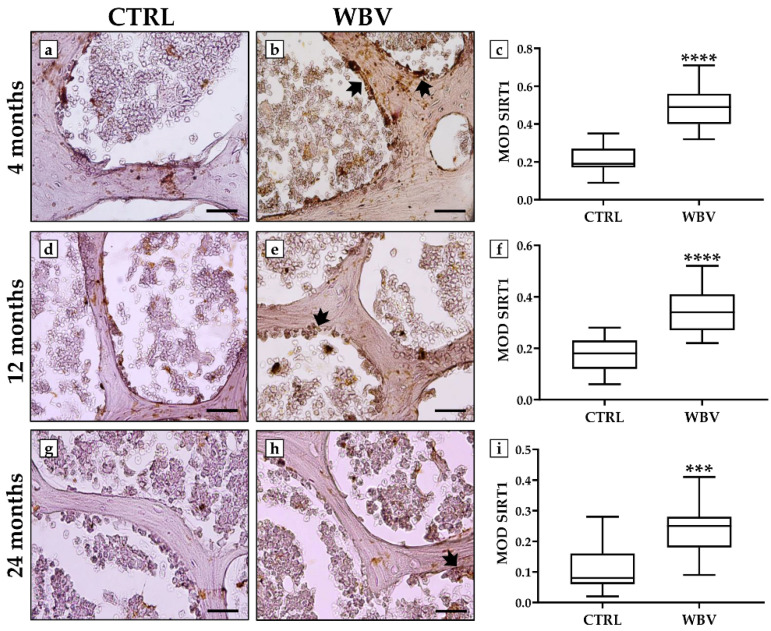
Evaluation of sirtuin 1 (SIRT1) immunolocalization in bone tissue of young, adult, and aged mice by immunohistochemistry analysis. (**a**–**c**) Bone tissue of young, trained mice showed the highest levels of SIRT1 (arrows), with a significant increase in its distribution compared with the CTRL group (**** *p* < 0.0001). (**d**–**f**) A significant increase in SIRT1 distribution (arrow) was observed in 12-month-old adult mice after WBV training compared with sedentary animals (**** *p* < 0.0001). (**g**–**i**) The distribution of SIRT1 (arrow) was significantly increased in the bone tissue of 24-month-old trained mice compared with the CTRL group (*** *p* < 0.001). For 20× images, the scale bar represents 100 μm. For each condition, the experiment was conducted in triplicate (n = 15 from N = 5 experiments).

Figure 5 shows a significant increase in SIRT1 distribution after WBV exposure, while reduced levels of this protein were observed in all sedentary groups. Specifically, in the 4-month-old young mice, the MOD values for SIRT1 were 0.21 ± 0.02 in the CTRL group and 0.48 ± 0.03 in the WBV group (*p* < 0.0001). In the 12-month-old adult mice, the MOD values for SIRT1 were 0.17 ± 0.02 in the CTRL group and 0.35 ± 0.02 in the WBV group (*p* < 0.0001), while in the 24-month-old aged mice, the MOD values for SIRT1 were 0.11 ± 0.02 in the CTRL group and 0.24 ± 0.02 in the WBV group (*p* < 0.001).

## 4. Discussion

Our study aimed to investigate the effects of an appropriately designed WBV protocol on the bone architecture of young, adult, and aged mice, as well as on the tissue distribution of potential markers of bone health, such as FNDC5, NOX4, and SIRT1, by identifying any alterations that might reflect age-related adaptation or degeneration processes and/or exposure to mechanical vibration. Particularly, we focused on these biomarkers since they are widely recognized for their involvement in key processes of regulation of bone and muscle metabolism, as well as in response to oxidative and mechanical stress [41,42,43]. Therefore, although the influence of WBV on the expression of FNDC5, NOX4, and SIRT1 has not been investigated or only partially, their relevance in musculoskeletal adaptation mechanisms makes them useful indicators to evaluate potential systemic and local effects resulting from exposure to mechanical vibrations.

Importantly, animals subjected to WBV showed a significant increase in body weight and profound changes in bone microarchitecture, with a significant increase in bone volume and trabecular thickness and a significant reduction in trabecular separation. These results agree with those of Wenger et al. (2010), who evaluated the efficacy of two WBV protocols, one at 0.5 g and the other at 1.5 g, both characterized by a vibration frequency of 32 Hz and administered for 30 min a day, 5 days a week for 3 months, in an 18-month-old mouse model. At the end of the training, both groups of animals showed a significant increase in bone density in the femoral head and neck, a reduction in collagen degradation markers, and an improvement in bone mineralization [44]. However, Lynch and colleagues found no significant changes in the skeletal structure of 7- and 22-month-old BALB/c mice subjected to a vibratory training protocol of 15 min per day, 5 days per week for 5 weeks, concluding that WBV does not slow the onset and/or progression of age-related bone loss [45]. Interestingly, this protocol was characterized by a vibration frequency of 90 Hz, confirming that the potential benefits of mechanical vibration are obtained at lower vibration frequencies and with longer recovery times [17,46]. In agreement, Lin et al. (2015) subjected 15-month-old C57BL/6 mice to two different WBV protocols, one at a relatively low frequency (5.6 Hz, 2 mm, 0.13 g) and one at a higher vibration frequency (13 Hz, 2 mm, 0.68 Hz), for 15 min per day, 5 days per week for 4 weeks, finding a marked improvement in muscle morphology, performance, and grip strength of the forelimbs of all animals. Noteworthy, a drastic reduction in inter-fiber adipose tissue was also found in the group subjected to vibration at a frequency of 13 Hz, highlighting the importance of strict control of all vibration parameters to effectively counteract the alterations that characterize musculoskeletal tissue during aging [47].

In our previous work, we suggested FNDC5, a transmembrane glycoprotein responsible for musculoskeletal adaptations to exercise, as a potential mediator of the beneficial effects of WBV on bone and muscle tissue in 4-month-old young mice [9]. Indeed, the expression of this protein and the polypeptide generated by its cleavage, irisin, is known to increase abundantly during exercise in skeletal muscle and bone, improving exercise capacity and bone formation [48]. Surprisingly, our results show a significant increase in FNDC5 distribution in the bone tissue of all trained groups, strengthening our hypothesis on the role of this protein in WBV-promoted musculoskeletal health. Although there is little evidence regarding the FNDC5 involvement in musculoskeletal adaptations to mechanical vibration, Huh and colleagues found a significant increase in circulating irisin after acute WBV administration in healthy females subjected to 6 weeks of twice-weekly WBV. Specifically, circulating irisin levels increased by 9.5% after the first session and 18.1% after the last training session, suggesting its involvement in physiological responses to WBV [12].

Several studies have pointed to irisin as a potential agent to counteract age-related musculoskeletal diseases due to its ability to upregulate the FNDC5 precursor in skeletal muscle and its osteoinductive power [18,49,50]. The NOX4 reduction we observed in bone could indicate improved musculoskeletal health. Indeed, pathologies that cause a progressive loss of muscle mass, such as muscular dystrophies, are characterized by an over-expression of NOX4, the inhibition of which produces the restoration of muscle-specific stem cells and, consequently, of regenerative potential [51]. NOX4 is also an important source of ROS, which promotes the sarcopenia onset, suggesting that NADPH oxidase is a potential therapeutic target to counteract age-related muscle atrophy [52]. Its involvement has also been proposed in osteoporotic bone loss, as its ROS would be responsible for osteoclastic activation and subsequent bone resorption. Indeed, NOX4-deficient mice are characterized by increased bone density and trabecular thickness, suggesting this enzyme is a potential therapeutic target to counteract osteoporosis [53,54].

In association with NOX4 reduction, bone tissue of all trained animals showed a significant increase in SIRT1 distribution, indicating its role in physiological adaptations to mechanical vibration. Indeed, SIRT1 is an essential regulator of inflammation, apoptosis, cellular senescence, mitochondrial biogenesis, and oxidative stress, representing a key determinant of aging in terms of lifespan and health [55]. Furthermore, SIRT1 expression increases during exercise, strengthening muscle mass, improving satellite cell activity, and counteracting sarcopenia and fragility [56]. Similarly, SIRT1 has been suggested as a promising regulator of bone homeostasis that maintains the balance between bone formation and resorption by regulating the ratio of osteoblasts to osteoclasts [57]. Preclinical studies showed that mice treated with SIRT1 agonists exhibited bone loss resistance in different osteoporosis models, suggesting SIRT1 as an attractive pharmacological target to counteract bone loss in aging [58].

Based on this evidence, we hypothesize that the administration of an appropriately designed WBV protocol in terms of vibration frequency, vibration exposure time, and recovery time can promote significant improvements in musculoskeletal health through up-regulation of FNDC5 and SIRT1 and down-regulation of NOX4. Although the mechanisms by which WBV influences the expression of these markers are not known, their regulation in response to mechanical vibrations appears to be associated with improvements in osteoblastic function, which could promote a better balance between bone formation and resorption. This could explain the observed changes in bone architecture, such as increased bone volume and trabecular thickness, which are indicators of more robust and resistant bone remodeling in response to exposure to mechanical vibrations. Therefore, an individually tailored vibratory protocol could improve current therapies to counter musculoskeletal decline by providing a viable alternative to exercise in elderly and/or sedentary subjects. Furthermore, the implementation of such protocols could be facilitated by adaptable and easily accessible tools, opening new opportunities for practitioners and clinicians in the creation of targeted interventions. These protocols could be further optimized through a multidisciplinary approach, integrating scientific knowledge on vibration parameters with individual needs, ensuring greater safety and effectiveness. In perspective, this could contribute to improving the quality of life of these individuals by promoting personalized interventions in both clinical and home settings. Undoubtedly, further studies are needed to investigate the effects of vibratory training programs on bone tissue through the involvement of the SIRT1-NOX4 axis, which is known to regulate oxidative stress and bone remodeling, as well as FNDC5 and irisin, which could influence mitochondrial biogenesis and muscle adaptation by regulating PGC-1α expression. In addition, an investigation of the serum levels of these molecules would be of interest to correlate systemic responses to vibratory training, helping to optimize vibratory protocols and improve their therapeutic effects.

## 5. Limits of the Study

The potential involvement of the SIRT1-NOX4 axis and FNDC5 in adaptations to WBV was shown for the first time in this study. Although our results are interesting and promising, further investigation will be needed to assess the role of these markers and their respective pathways involved in musculoskeletal responses to mechanical vibration. Mainly, quantitative analyses could be performed with investigations of serum markers to study the potential systemic effects of WBV. Another limitation of this study is the small sample size, which could affect the robustness and reproducibility of the results. Future studies with larger samples will be critical to confirm our results.

## 6. Conclusions

Musculoskeletal decline is a condition that afflicts the elderly and individuals forced into a sedentary lifestyle, predisposing them to degenerative diseases such as osteoporosis and sarcopenia. Such conditions not only impair the quality of life but significantly increase the risk of fractures and disability. Considering many older or physically limited individuals’ difficulties in maintaining a regular exercise routine, vibratory training protocols offer a promising alternative to promote musculoskeletal health.

WBV protocols, when properly designed and tailored to individual needs, can stimulate bone tissue and positively influence several biomarkers involved in regulating musculoskeletal health. Among these, the NOX4-SIRT1 axis could be an essential mediator of the beneficial effects of vibration on bone tissue. Activation of this axis has shown positive effects on bone tissue, suggesting that WBV may lead to relevant structural and molecular changes that can counteract age- or inactivity-related musculoskeletal decline.

The differences observed between previous evidence and our study can be attributed to several factors, such as the experimental design, the age of the animals, and the specific vibration parameters used, including frequency, acceleration, and session duration. Furthermore, when applying WBV protocols to humans, factors such as dietary intake, baseline physical activity levels, and genetic predispositions should be considered, as they may significantly influence the response of the musculoskeletal and metabolic system to mechanical vibration. These aspects, often variable in the human population, underline the need to design personalized protocols to maximize the benefits of WBV. Future research should not only aim to optimize WBV parameters to achieve the best clinical outcomes but also explore how biological and environmental factors may modulate the efficacy of such interventions. In addition, further studies should investigate the effect of WBV on other molecular pathways involved in bone response for a deeper understanding of physiological adaptations to mechanical vibration.

## Data Availability

The data presented in this study are available on request from the corresponding author.

## Supplementary Materials

The following supporting information can be downloaded at https://www.mdpi.com/article/10.3390/jfmk10010026/s1: Figure S1: Subdivision of animals used in this study; Figure S2: Measurement of body weight in sedentary murine models of different age groups; Figure S3: Morphometric analysis of bone tissue from sedentary mice; Figure S4: Immunohistochemical analysis for fibronectin type III domain-containing protein 5 (FNDC5), NADPH oxidase 4 (NOX4), and sirtuin 1 (SIRT1) in bone tissue of sedentary mouse.
