# Supplementary material for: Bone Adaptations to a Whole Body Vibration Protocol in Murine Models of Different Ages: A Preliminary Study on Structural Changes and Biomarker Evaluation
Source: J Funct Morphol Kinesiol. 2025 Jan 10;10(1):26. doi: 10.3390/jfmk10010026 (PMC11755639; doi:10.3390/jfmk10010026)
Supplement: Supplementary file 1 [file jfmk-10-00026-s001.zip › jfmk-3378823-supplementary.pdf]

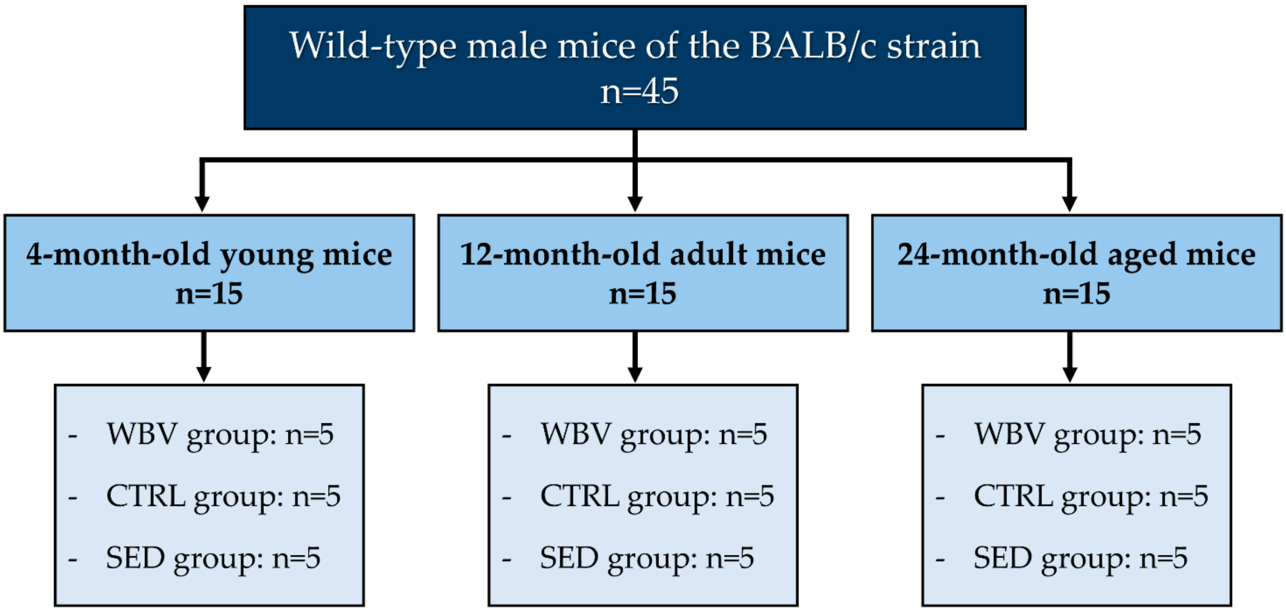

Figure S1. Subdivision of animals used in the study. A total of 45 male mice belonging to the BALB/c strain were used and divided into three groups according to age: 4-month-old young mice (n=15), 12-month-old adult mice (n=15) and 24-month-old aged mice (n=15). For each age group, three groups were considered: an intervention group subjected to a specific whole body vibration (WBV) protocol (n=5); a control group (CTRL) consisting of mice subjected to the same regimen of placement on the box in the platform, the same environmental exposure including motor sounds, but not exposed to vibratory training (n=5); and an additional control group (SED) with sedentary mice not subjected to any training (n=5).

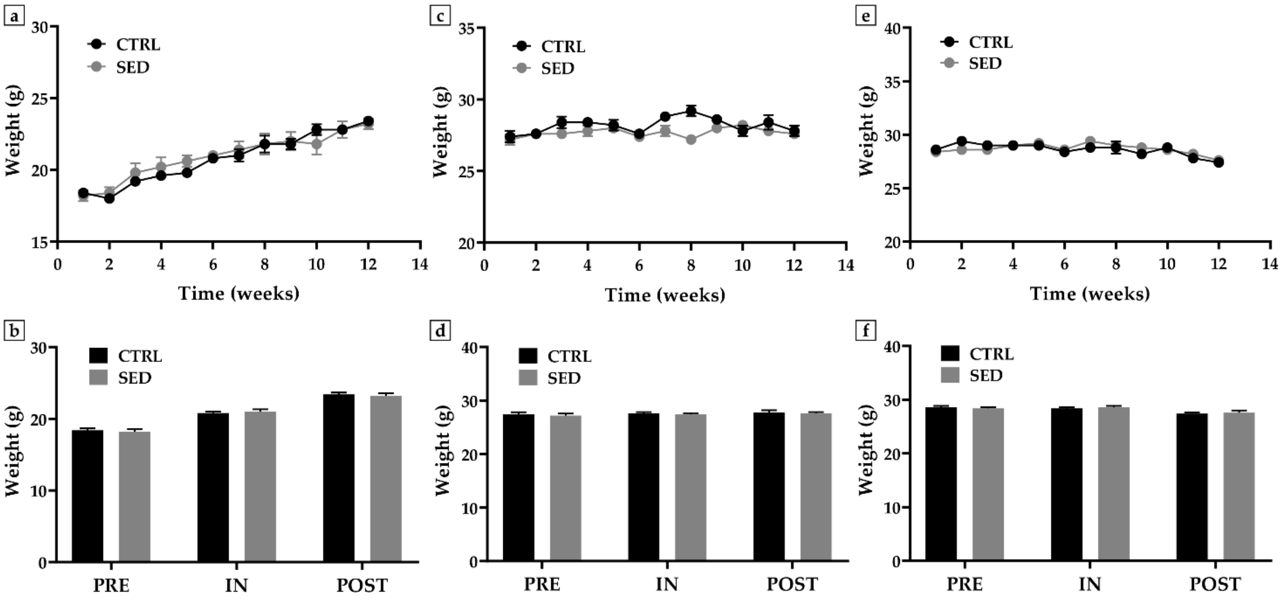

Figure S2. Measurement of body weight in sedentary murine models of different age groups. (a) Weight change from week 1 to week 12 in 4-month-old young mice. (b) Mean weight measurement at week 1 (PRE phase), week 6 (IN phase), and week 12 (POST phase) in 4-month-old young mice. PRE phase: 18.4 ± 0.2 for the CTRL group vs. 18.2 ± 0.4 for the SED group. IN phase: 20.8 ± 0.2 for CTRL group vs 21.0 ± 0.3 for SED group. POST phase: 23.4 ± 0.2 for the CTRL group vs. 23.2 ± 0.4 for the SED group. (c) Weight change from week 1 to week 12 in 12-month-old adult mice. (d) Measurement of mean weight at PRE, IN, and POST phases in 12-month-old adult mice. PRE phase: 27.4 ± 0.4 for the CTRL group vs 27.2 ± 0.4 for the SED group. IN phase: 27.6 ± 0.2 for CTRL group vs 27.4 ± 0.2 for SED group. POST phase: 27.8 ± 0.4 for the CTRL group vs. 27.6 ± 0.2 for the SED group. (e) Weight change from week 1 to week 12 in 24-month-old aged mice. (f) Measurement of mean weight at PRE, IN, and POST phases in 24-month-old aged mice. PRE phase: 28.6 ± 0.2 for the CTRL group vs. 28.4 ± 0.2 for the SED group. IN phase: 28.4 ± 0.2 for CTRL group vs 28.6 ± 0.2 for SED group. POST phase: 27.4 ± 0.2 for the CTRL group vs. 27.6 ± 0.4 for the SED group.

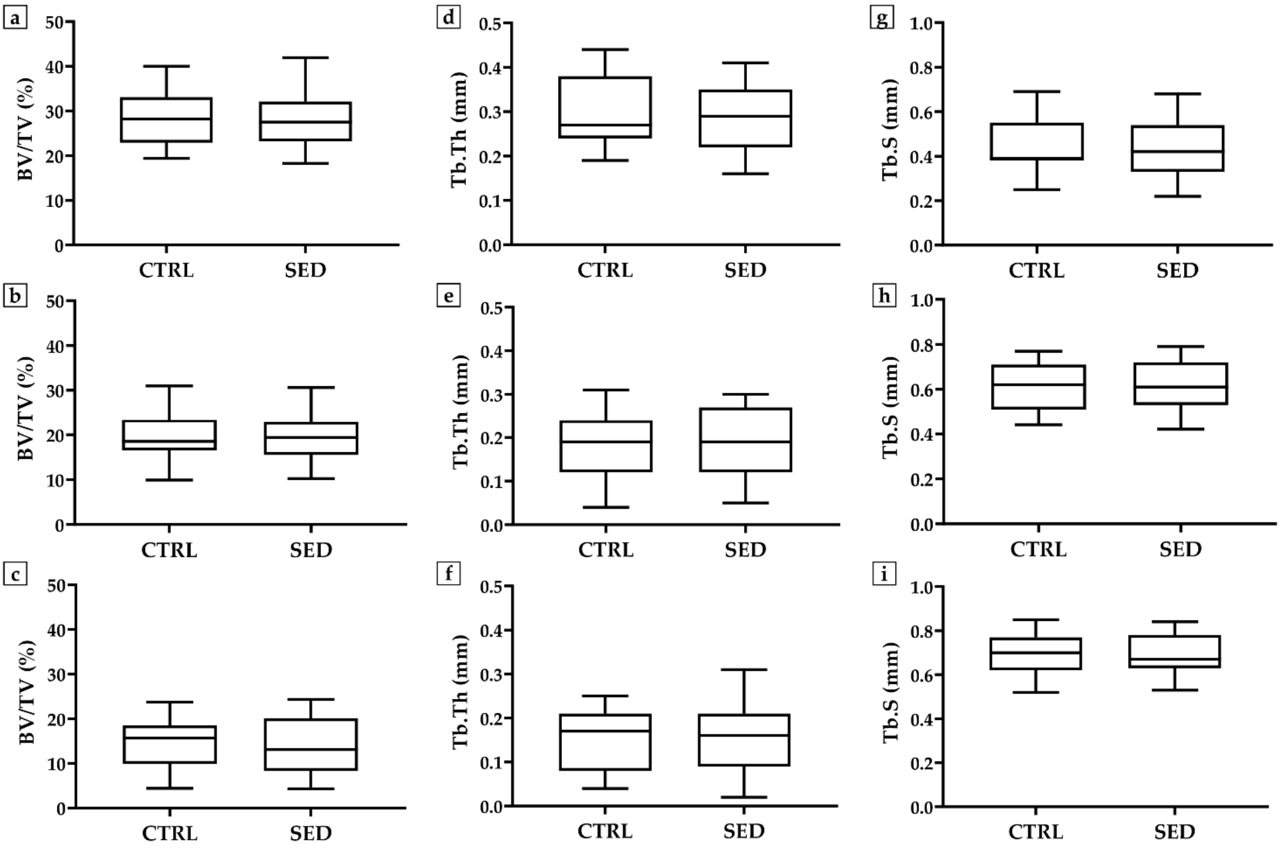

Figure S3. Morphometric analysis of bone tissue from sedentary mice. (a) Bone volume (BV/TV) values in 4-month-old young mice: 28.2 ± 1.5 for the CTRL group vs 28.0 ± 1.6 for the SED group (p=0.93). (b) BV/TV values in 12-month-old adult mice: 19.7 ± 1.5 for the CTRL group vs. 19.4 ± 1.3 for the SED group. (b) BV/TV values in 12-month-old adult mice: 19.7 ± 1.5 for the CTRL group vs. 19.4 ± 1.3 for the SED group (p=0.91). (c) BV/TV values in 24-month-old aged mice: 14.6 ± 1.4 for the CTRL group vs 13.9 ± 1.7 for the SED group (p=0.74). (d) Trabecular thickness (Tb.Th) values in 4-month-old young mice: 0.29 ± 0.02 for the CTRL group vs 0.29 ± 0.02 for the SED group (p=0.98). (e) Tb.Th values in 12-month-old adult mice were 0.18 ± 0.02 for the CTRL group vs. 0.19 ± 0.02 for the SED group (p=0.78). (f) Tb.Th values in 24-month-old aged mice were 0.15 ± 0.02 for the CTRL group vs. 0.15 ± 0.01 for the SED group (p=0.90). (g) Trabecular separations (Tb.S) values in 4-month-old young mice: 0.45 ± 0.03 for the CTRL group vs 0.44 ± 0.03 for the SED group (p=0.84). (h) Tb.S values in 12-month-old adult mice: 0.61 ± 0.03 for CTRL group vs 0.61 ± 0.02 for SED group (p=0.87). (i) Tb.S values in 24-month-old aged mice: 0.69 ± 0.02 for the CTRL group vs 0.68 ± 0.02 for the SED group (p=0.81).

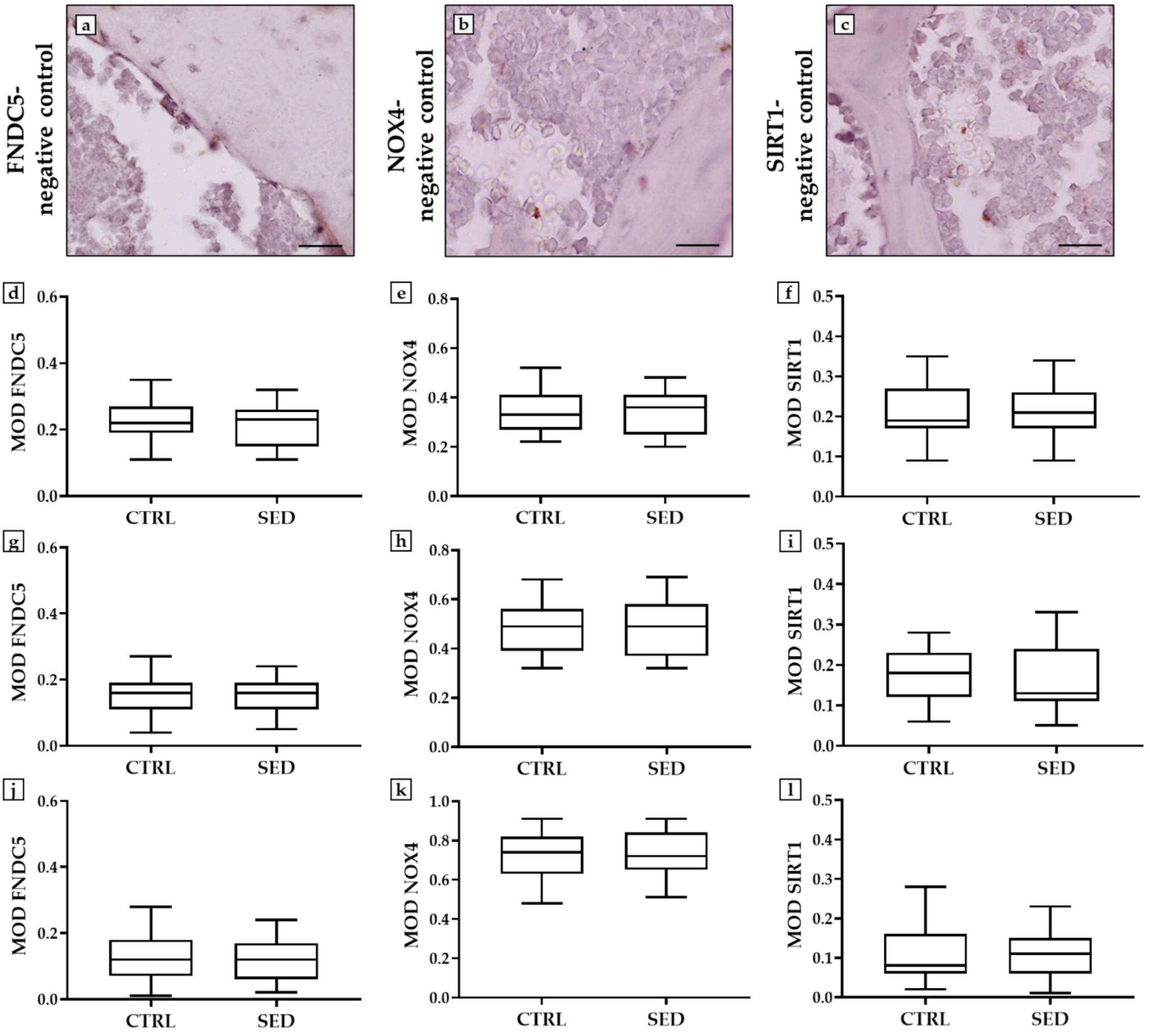

Figure S4. Immunohistochemical analysis for fibronectin type III domain-containing protein 5 (FNDC5), NADPH oxidase 4 (NOX4), and sirtuin 1 (SIRT1) in bone tissue of sedentary mouse. (a) FNDC5-negative control in bone tissue. (b) NOX4-negative control in bone tissue. (c) SIRT1-negative control in bone tissue. 40× images, scale bar represents 50 µm. (d) Mean optical density (MOD) values for FNDC5 in 4-month-old young mice: 0.22 ± 0.02 for the CTRL group vs 0.21 ± 0.02 for the SED group ($p=0.74$). (e) MOD values for NOX4 in 4-month-old young mice: 0.33 ± 0.02 for CTRL group vs 0.34 ± 0.02 for SED group ($p=0.76$). (f) MOD values for SIRT1 in 4-month-old young mice: 0.21 ± 0.02 for CTRL group vs 0.22 ± 0.02 for SED group ($p=0.89$). (g) MOD values for FNDC5 in 12-month-old adult mice: 0.15 ± 0.02 for CTRL group vs 0.15 ± 0.01 for SED group ($p=0.86$). (h) MOD values for NOX4 in 12-month-old adult mice: 0.47 ± 0.03 for CTRL group vs 0.48 ± 0.03 for SED group ($p=0.88$). (i) MOD values for SIRT1 in 12-month-old adult mice: 0.17 ± 0.02 for CTRL group vs 0.16 ± 0.02 for SED group ($p=0.79$). (j) MOD values for FNDC5 in 24-month-old aged mice: 0.12 ± 0.02 for CTRL group vs 0.12 ± 0.02 for SED group ($p=0.94$). (k) MOD values for NOX4 in 24-month-old aged mice: 0.73 ± 0.03 for CTRL group vs 0.73 ± 0.03 for SED group ($p=0.89$). (l) MOD values for SIRT1 in 24-month-old aged mice: 0.11 ± 0.02 for CTRL group vs 0.10 ± 0.01 for SED group ($p>0.99$).
